# A Review on Energy Management System for Grid‐Connected Utility‐Scale Renewable Hybrid Power Plants

**DOI:** 10.1002/wene.70004

**Published:** 2025-03-11

**Authors:** Rujie Zhu, Kaushik Das, Poul Ejnar Sørensen, Anca Daniela Hansen

**Affiliations:** ^1^ DTU Wind Technical University of Denmark Roskilde Denmark

**Keywords:** electricity markets, energy management system, hybrid power plant, uncertainty

## Abstract

In recent years, renewable hybrid power plants (HPPs) have experienced rapid expansion. Energy management systems (EMSs) are vital to these facilities, helping maximize economic returns for owners and shaping operational strategies across various time scales. However, a comprehensive review of advancements in this field is still lacking. This paper presents an in‐depth analysis of EMS research tailored for grid‐connected, utility‐scale renewable HPPs. It begins by outlining common HPP configurations, which form the foundation for EMS modeling. Five key EMS approaches are then discussed in detail, namely, rule‐based methods, mathematical optimization, model predictive control, deep reinforcement learning, and stochastic dynamic programming. Following that, the paper categorizes the types of market participation and uncertainties addressed by EMS, and it introduces several industrial EMS tools. Finally, the discussion highlights existing gaps in EMS research for HPPs. Overall, this paper provides cutting‐edge insights into EMS for HPPs, serving as a valuable resource for both researchers and industry professionals involved in HPP EMS development.

## Graphical abstract

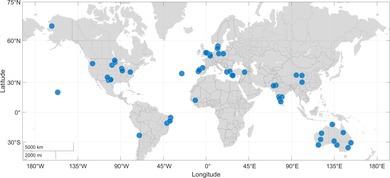

**Abbreviations**
BESS: battery energy storage system
CAES: compressed air energy storage
CSP: concentrated solar power
CVaR: conditional value‐at‐risk
DO: deterministic optimization
DRCC: distributionally robust chance constraint
DRL: deep reinforcement learning
DRO: distributionally robust optimization
EMS: energy management system
HES: hybrid energy system
HPP: hybrid power plant
IEA: International Energy Agency
ISO: independent system operator
MDP: Markov decision process
MG: microgrid
MILP: mixed integer linear programming
MO: mathematical optimization
MPC: model predictive control
PV: photovoltaic
RES: renewable energy source
RO: robust optimization
RTO: regional transmission organization
SDP: stochastic dynamic programming
SO: stochastic optimization
SPP: solar power plant
VaB: value‐at‐best
VaR: value‐at‐risk
VPP: virtual power plant
WPP: wind power plant

## 1 Introduction

The increasing adoption of wind, solar, and storage technologies, supported by favorable policies and cost reductions, has enabled renewable energy sources (RESs) to become reliable sources of electricity. However, weather‐dependent RESs have become one of the main challenges to the operation and control of energy systems. These challenges include the energy system balancing caused by the variable and uncertain generation from wind and solar, the need for infrastructure upgrades to accommodate the large‐scale RES integration, the need for effective market mechanisms to support the integration, and so on.

Hybrid power plants (HPPs), as a new generation system (Asbeck 2018), have garnered attention worldwide in both industry (Gamesa 2022; Bolinger et al. 2023) and academia (Montañés et al. 2022; Das et al. 2019; Hansen et al. 2021). Despite their growing importance, a standardized definition for HPPs is still lacking. Table 1 summarizes the definitions of HPPs from different institutions. As seen from the table, the common feature of HPPs is that they combine two or more sources of power generation and/or energy storage, such as solar, wind, hydro, battery, and so on. HPPs are co‐located facilities that integrate multiple types of generation assets, all within a single geographical location. These assets are typically owned and managed by one company, allowing for centralized operation and efficient coordination. In contrast, virtual power plants (VPPs) aggregate several distributed energy resources that are not co‐located, with assets spread across various locations. The ownership of these assets is often diverse, with different entities owning individual components, while an aggregator coordinates their operation through a sophisticated digital platform. The main motivation for using HPPs is to overcome the variability and uncertainty of renewable energy sources and to provide more reliable, affordable, and sustainable power supplies. The foreseen advantages of HPPs are:

- From society's viewpoint, HPPs can make more efficient use of available land by sharing electrical infrastructure, which boosts both installed capacity and energy production per unit area (WindEurope 2019). Additionally, more renewable energy can be integrated into grids with limited grid connection capacity through overplanting. Finally, the flexibility of HPP generation supports the phase‐out of fossil fuel‐based generators.
- From costs' viewpoint: Co‐locating facilities, power conversion units, and other plant components can significantly lower the capital expenditures required for infrastructure (Gorman et al. 2020).
- From power systems' viewpoint: By incorporating energy storage and leveraging the naturally offsetting production profiles of wind and solar power (Widén 2011), HPPs can achieve higher capacity factors and more firm power outputs, thereby reducing balancing needs and renewable curtailments. Furthermore, in the long run, overplanting can postpone the need for transmission upgrades as renewable penetration expands.
- From HPP owners' viewpoint: With the benefits brought by storage, HPPs can obtain more revenue streams by participating in multiple electricity markets, such as ancillary service markets.
- From HPP developers' viewpoint: developers can streamline the development, permitting, operation, and maintenance processes. This integrated approach eliminates the need to repeat these processes separately for each technology, thus enhancing efficiency and reducing duplication of efforts (Gorman et al. 2020).

**TABLE 1:** Overview of different definitions of hybrid power plants.

Institution and reference	Definition
National Renewable Energy Laboratory (NREL) (Dyke et al. 2020)	“Hybrid power plants are power plants that contain two or more technologies that may potentially include wind turbines, solar photovoltaic (PV), concentrated solar power (CSP), storage, geothermal power, hydropower, biomass, natural gas, oil, coal, or nuclear power. These hybrid plants can be used to generate electricity or other products such as hydrogen”
The Energy Systems Integration Group (Ahlstorm et al. 2019)	“A hybrid power plant is a combination of multiple technologies that are physically and electronically controlled by the hybrid owner/operator behind the point of interconnection and offered to the grid operator (or to some other customer) as a single resource.”
WindEurope (WindEurope 2019)	“A hybrid power plant is a power‐generating facility that converts primary energy into electrical energy and which consists of more than one power‐generating module connected to a network at one connection point”
International Energy Agency (IEA) Wind TCP Task 50 ((IEA 2022)	“A hybrid power plant is a combination of two or more electricity generation and/or two or more storage technologies used to provide electrical power services that can be coordinated at a single connection point with either unidirectional or bi‐directional power flow”

**FIGURE 1:**
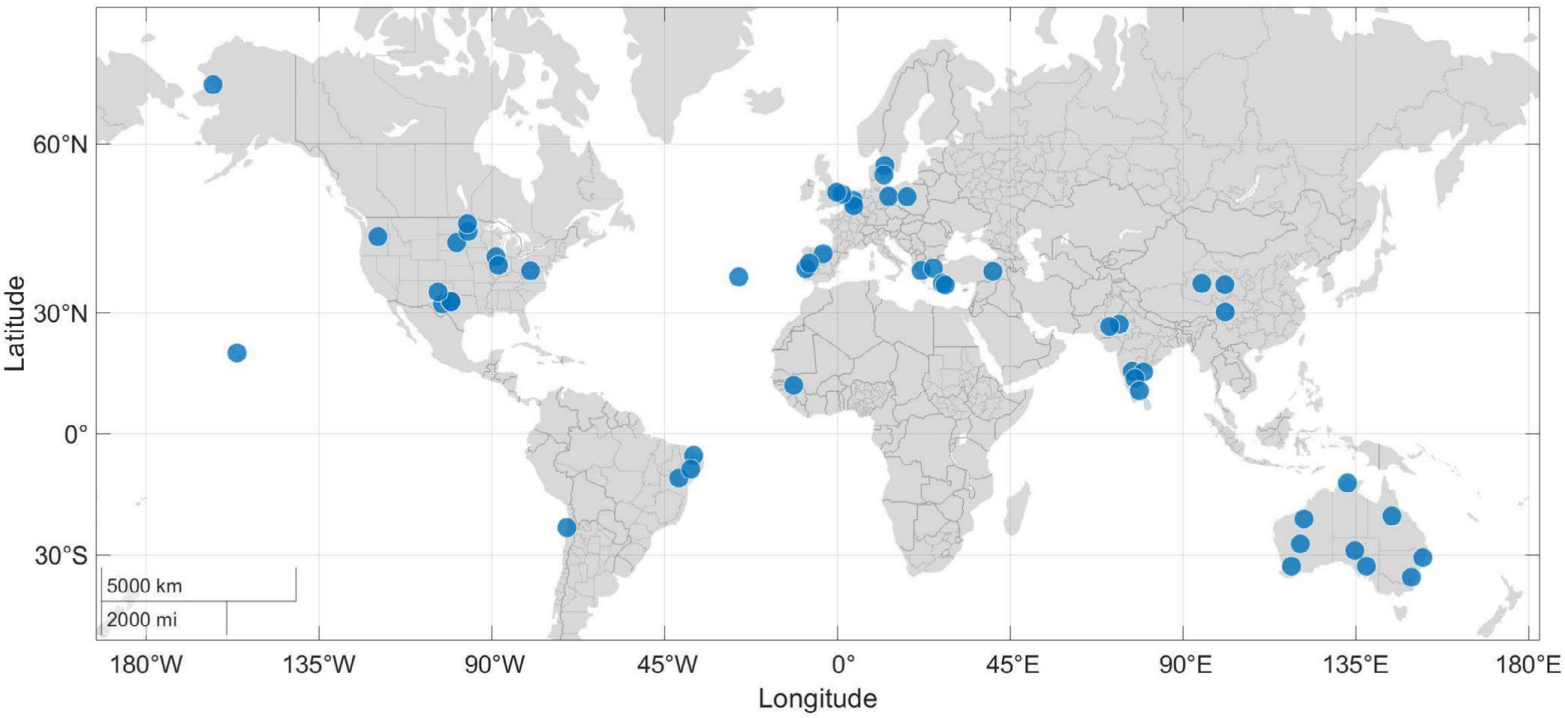
Global operating and pipelined renewable hybrid power plants (Incomplete statistics).

The Kennedy Energy Park, recognized as the world's first utility‐scale HPP, was connected to the Australian power grid in 2019. By the middle of 2023, the global operating and pipelined renewable hybrid power plants have significantly increased (Bolinger et al. 2023; WindEurope 2022), as depicted in Figure 1. Countries such as India (Das, Jani, et al. 2020) and Brazil (Andra Santos et al. 2020) have drafted policies for integrating HPP into grids. Leading commercial enterprises have unveiled innovative hybrid power solutions aimed at enhancing the design and operational efficiency of HPPs (Petersen et al. 2018). In academia, the focuses of HPPs have been on sizing and physical design (Stanley and King 2022), optimal energy management (Zhu et al. 2022), supervisory control (Long et al. 2021; Long, Das, and Sørensen 2022), and so on. Despite the promising potential of HPPs, their economic value, ability to provide grid services, and other key aspects remain insufficiently understood. Task 50 of the IEA Wind TCP initiative was introduced to foster global collaboration in advancing hybrid wind power plants, with a primary emphasis on optimizing their design and operational strategies. The IEA Wind TCP Task 50 highlighted three primary challenges in developing HPPs: demonstrating economic viability, the absence of reliable validation data, and the need for advanced optimization and control tools (IEA 2022). Among these, energy management systems (EMS) stand out as pivotal tools for assessing the economic potential of HPPs. Consequently, EMS research has gained significant traction in recent years.

**FIGURE 2:**
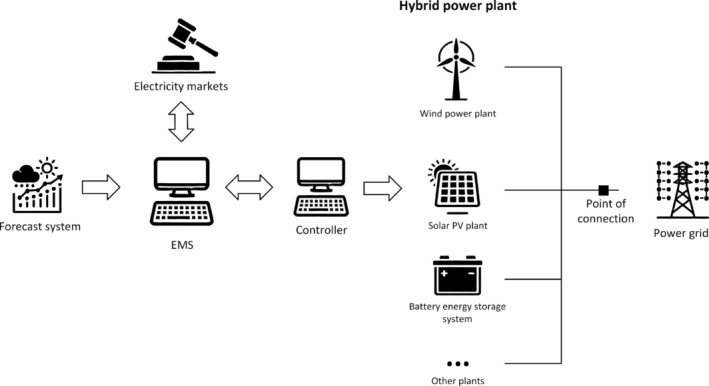
Diagram of operation and control of hybrid power plants.

Figure 2 demonstrates the diagram of the operation and control of HPPs. The EMS is the central component of HPPs, interfacing with electricity markets, integrating weather forecasts, and incorporating market, asset, and grid information to optimize plant operation. EMS can be categorized according to its integration with the controller, which may either be integrated directly into the controllers or function independently. Integrated EMS works closely with the plant's control systems, enabling real‐time adjustments and enhanced coordination of various components. Conversely, independent EMS leverages advanced algorithms and data processing capabilities to implement sophisticated and complex features, though they may have less direct control over immediate plant operations. The EMS discussed in this review paper primarily belongs to the latter category. One of the EMS's core responsibilities is to determine and implement optimal strategies for HPP generation that conform to various market requirements or contractual agreements. At the same time, it manages uncertainties to enhance overall energy performance and boost profitability across multiple operating horizons. In addition, the EMS provides supervisory guidance to the HPP controller, which then applies the setpoints derived from the EMS (Long, Das, Pombo, and Sørensen 2022).

Naval and Yusta (2021) provide a structured examination of VPP models that operate across multiple energy markets. Their analysis encompasses the theoretical formulations, the optimization techniques used to solve them, strategies for market engagement, and validation through real‐world case studies. Yu et al. (2019) review the uncertainties associated with VPP and the relevant optimization approaches to model those uncertainties. The real‐world VPP projects are also discussed. Sharma et al. (2022) introduce a comprehensive framework for classifying microgrid (MG) energy management strategies, organized according to supervisory control layers, operational time scales, and decision‐making approaches. The optimization‐based and power control‐based approaches are further summarized. Ma, Li, and Zhang (2022) present and review the typical architecture of multi‐microgrids, including the physical, information, and application layers. It also critically analyzes the challenges in uncertainty modeling and solutions in multi‐microgrids. Yang et al. (2022) review the optimal energy management of battery storage in hybrid energy systems (HESs), including the introduction of battery models, the analysis of energy management objectives, and the classification of optimization techniques. The self‐scheduling problem for generation companies using robust optimization considering price uncertainty is discussed by Vatani et al. (2018). The bidding strategies for renewable power producers in spot markets are reviewed by Peng et al. (2023). The power production models of wind and solar in hybrid wind‐solar power plants are analyzed by Lindberg et al. (2021).

In summary, existing review studies on EMS predominantly concentrate on VPPs, MGs, and HESs, leaving reviews on EMS for HPPs notably scarce and incomplete. Unlike MGs and HESs, which primarily focus on meeting critical demands locally, and VPPs, which aggregate distributed energy resources for optimized energy management and grid services, HPPs are designed to replace traditional power plants and act as generators connected to the main grid (Dyke et al. 2020). The objective of the EMS is to maximize value for HPP owners. Although there are similarities between the EMS of VPPs, MGs, and HESs and that of HPPs, the current literature does not encompass all the pertinent aspects of HPPs. For example, only one optimization methodology is discussed by Naval and Yusta (2021). Sharma et al. (2022) do not discuss the integration of MGs into electricity markets. Hence, a comprehensive and inclusive review of EMS for HPPs is imperative, addressing multifaceted aspects to bridge this critical gap.

**FIGURE 3:**
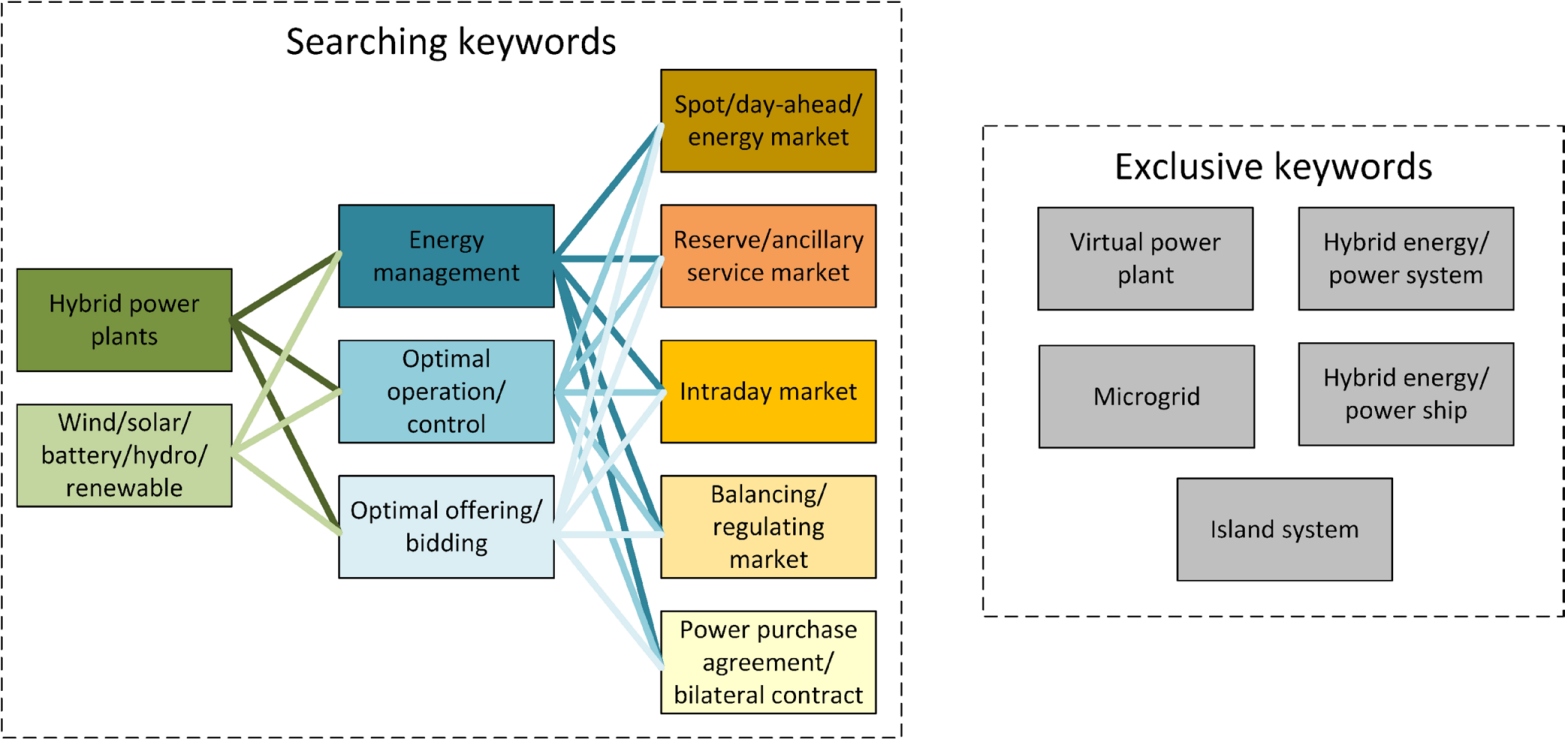
Keywords for searching, selection, and exclusion of the papers.

This paper presents a comprehensive review of existing research on EMS for HPPs. It explores HPP classifications, key features, mathematical approaches to EMS, market considerations, uncertainty factors, and industrial tools. The goal is to offer an in‐depth overview of EMS for utility‐scale, grid‐connected HPPs. This manuscript is developed from the first author's PhD. thesis (Zhu 2023). To ensure that the relevant articles are included, combinations of three groups of keywords are searched in Google Scholar, IEEE Xplore, ScienceDirect, and DTU Findit (the electronic library of the Technical University of Denmark), as shown in Figure 3. Given the broad spectrum of EMS applications, it is essential to define the boundaries of this review to maintain a concentrated discussion. This review will specifically exclude studies related to EMS for virtual power plants, hybrid energy/power systems, microgrids, island systems, and hybrid energy/power ships. Following or managing load profiles within a network is one of the primary purposes of these technologies. Including them would substantially broaden the scope, leading to an unwieldy number of studies that differ significantly in focus on HPPs. HPPs operate as distinct generation facilities with a direct connection to the power grid. This unique point of connection highlights the role of HPPs in active power generation rather than just load management, necessitating a separate and focused analysis of their EMSs. The period considered is from 2015 to 2023. Finally, after a detailed reading, there are a total of 127 papers included in this study.

The rest of the paper is structured as follows: Section 2 delves into the classification and configurations of HPPs. Section 3 examines and contrasts various EMS methodologies. Regional and national markets considered in EMS are reviewed in Section 4, while Section 5 focuses on uncertainty sources. Industrial EMS tools are explored in Section 6. Research gaps in EMS for HPPs are highlighted in Section 7, and Section 8 wraps up with the conclusions.

## 2 HPP Configuration

**FIGURE 4:**
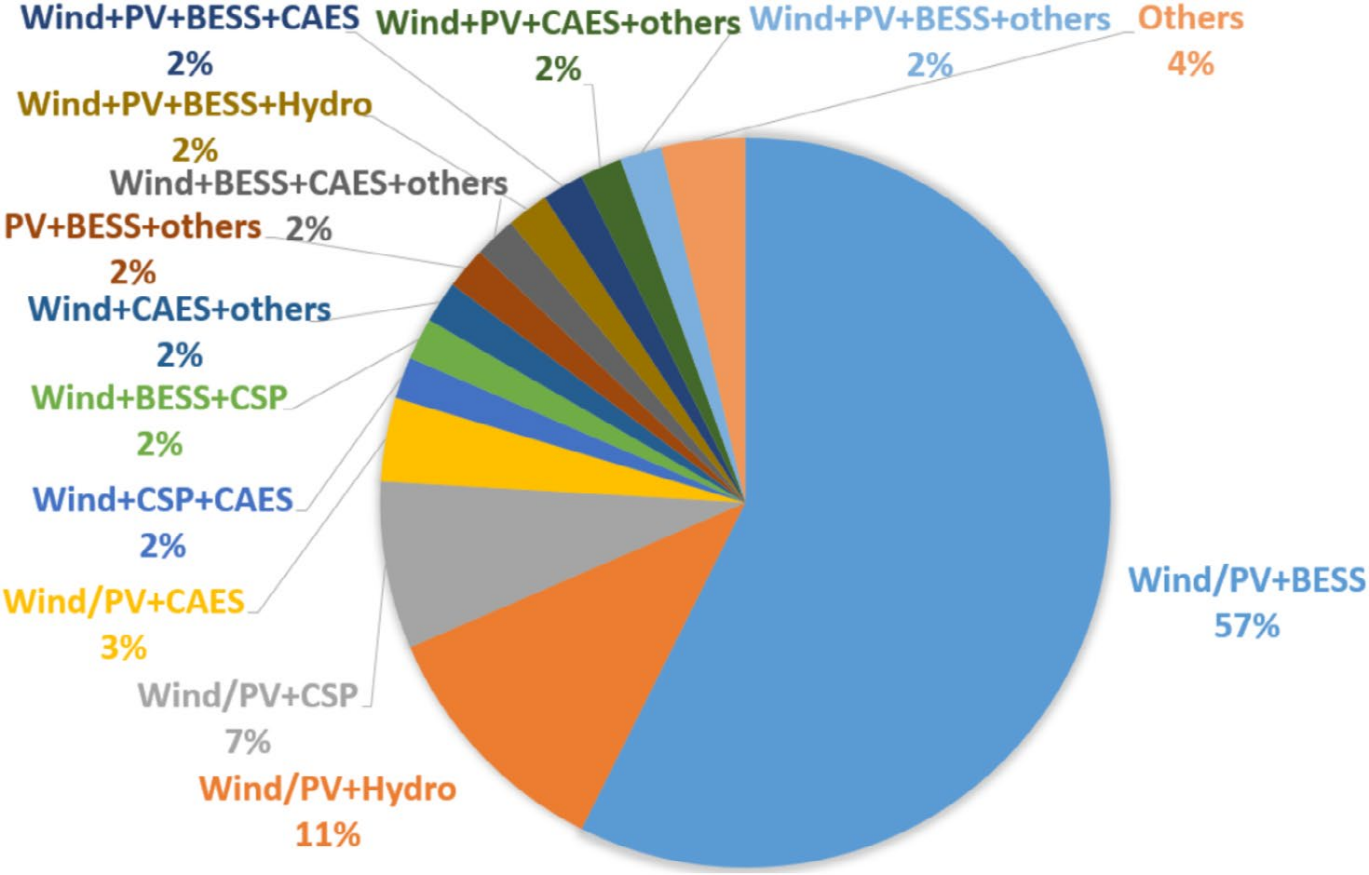
Overview of the proportion of different HPP configurations.

The configurations of HPPs are diverse, incorporating a wide range of assets, such as wind power plants (WPPs), solar PV plants (SPPs), battery energy storage systems (BESSs), concentrated solar plants (CSPs), and hydropower plants. Figure 4 illustrates the distribution of these configurations. Wind, PV, and BESS emerge as the most prominent technologies in EMS research for HPPs, while other technologies hold promise for integration into future designs. Among the configurations, wind/solar/BESS, wind/solar/hydro, and wind/solar/CSP stand out as the most prevalent.

### 2.1 Wind, PV, and BESS

In the research literature, WPP, SPP, and BESS combinations are frequently highlighted as the most effective configurations for HPPs. The synergy between WPP and SPP in HPPs can lead to a more stable power output than each technology operating independently. This is largely due to the often inverse relationship between solar and wind energy availability (Widén 2011). Moreover, integrating BESS into HPPs is increasingly seen as a strategic move. Batteries offer several advantages: they are cost‐effective, can be installed flexibly, respond rapidly to grid requirements, and boast high power and energy density. These characteristics make batteries an ideal storage solution to complement renewable energy sources. With such a combination, HPPs can generate more consistent and controllable power and stand to gain greater financial benefits in the electricity market. Including BESS enables HPPs to offer valuable ancillary services, such as power balancing and frequency support. When combined with effective energy management strategies that optimize the allocation of power and energy capacities, HPPs can significantly enhance their market profitability and operational efficiency.

### 2.2 Wind, PV, and Hydro

The combination of WPP, SPP, and hydropower plants is highly valued for its flexibility and quick regulation capabilities. Research in northeastern Italy demonstrates the natural synergy between water and solar energy (Francois et al. 2016; Li and Qiu 2016). Hydropower's ability to smooth out the fluctuating output of PV systems and provide nighttime compensation makes it essential for reducing short‐term generation variability. The Longyangxia PV‐hydropower HPP is a prime example of this enhanced dispatchability (Rogner 2015). In the medium to long term, PV systems can maintain capacity factors during dry years, thanks to the relatively stable annual solar energy output (An et al. 2015). As noted by Lee et al. (2020), there is also a growing interest in integrating floating solar PV with hydropower plants. This innovative approach not only reduces water evaporation by limiting airflow and solar absorption but also conserves water resources for future use.

### 2.3 Wind, PV, and CSP

While PV cells harness sunlight to generate electric current by moving electrons, CSP takes a different approach by converting solar energy into high‐temperature heat. This heat then powers engines and generators to produce electricity. One of the key advantages of CSP is its built‐in thermal energy storage system, which enables continuous power generation even when sunlight is scarce (Powell et al. 2017). This dispatchable nature makes CSP an attractive option for co‐location with WPP and SPP.

## 3 EMS Methodologies

**FIGURE 5:**
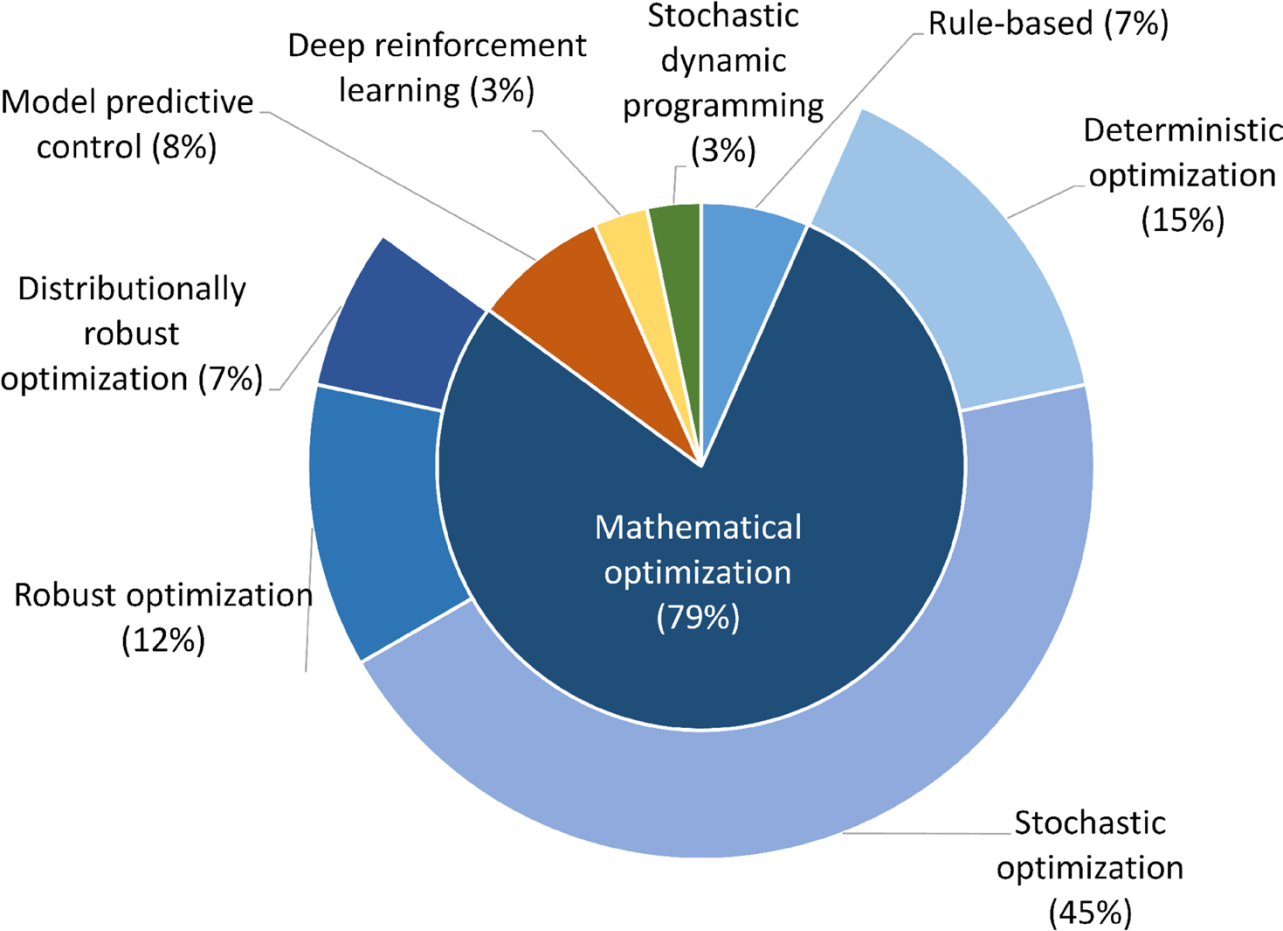
Overview of the proportion of different EMS methodologies.

In this section, we provide a detailed examination of the diverse approaches developed for energy management in HPPs. As illustrated in Figure 5, these approaches can be organized into five overarching categories: rule‐based, mathematical optimization (MO), model predictive control (MPC), deep reinforcement learning (DRL), and stochastic dynamic programming (SDP). Each category has its advantages and limitations in dealing with different aspects of the energy management problem. Among the five categories, MO‐based methodologies dominate the EMS research, with stochastic optimization (SO) holding the largest share. Other MO‐based methodologies, such as deterministic optimization (DO), robust optimization (RO), and distributional robust optimization (DRO), also contribute to addressing complex energy management challenges. This section will compare and contrast these methodologies and highlight their strengths and weaknesses.

### 3.1 Rule‐Based Approach

A straightforward approach to operate HPPs involves setting rules for the charging and discharging of storage as well as the curtailment of renewable energy. This is known as rule‐based EMS. In the literature, the rule‐based EMS can be classified as price‐driven rules for the market participation of HPPs and imbalance‐driven rules for the real‐time operation of HPPs. Price‐driven rules guide the storage to charge and discharge during low‐price and high‐price intervals, respectively. For example, Zhang et al. (2018) suggest a rule‐based EMS for HPPs with wind and battery to establish power schedules that align with time‐of‐use electricity pricing structures. The battery is used to shift forecasted wind energy from off‐peak and low‐price periods to high‐price periods to perform energy arbitrage.

**FIGURE 6:**
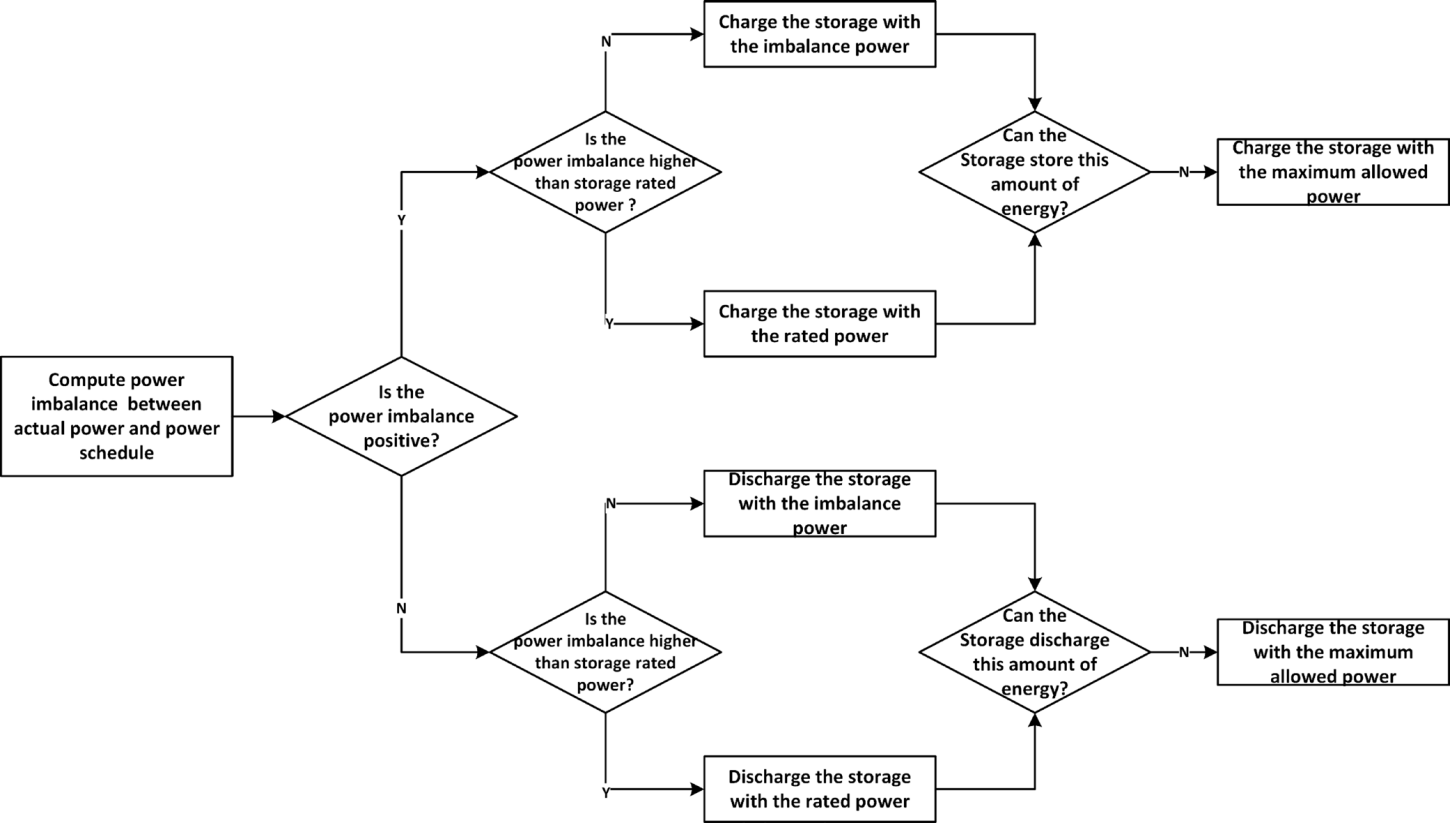
An example flowchart of the imbalance‐driven rule‐based EMS.

During real‐time operations, the rule‐based EMS is mainly designed to employ storage to mitigate power imbalance between the actual generation and the pre‐determined power schedules due to forecasting errors. Figure 6 showcases a flowchart of this rule‐based EMS as described by Das, Grapperon, et al. (2020). The flowchart starts with judging the power imbalance between actual power and the power schedule. Excess power must be charged into the storage if it is positive. The charging power depends on the value of imbalanced power, the rated power of storage, and the maximum allowed power constrained by the storage's energy capacity. Similarly, the same considerations apply if the power imbalance is negative. Zhang et al. (2016) propose a more complicated rule‐based EMS, where the batteries are divided into two groups to avoid the frequent charging/discharging state exchange. One group continuously charges until full, and the other discharges until empty, defining the asynchronous exchanging strategy where their operational states are independent. In contrast, the simultaneous exchanging strategy maintains opposite states between the groups at all times; when one group finishes discharging, the other, not necessarily fully charged, begins to discharge, ensuring coordinated and inverse operation. Their simulation finds that while the simultaneous strategy enhances battery performance in tracking power schedules, it negatively impacts economic performance. Conversely, the asynchronous strategy yields better economic outcomes.

Rule‐based EMS is simple to implement and does not require complex mathematical models, making it ideal for tasks with long computation times, for example, design and sizing of HPPs (Das et al. 2022). However, since it relies on predefined rules, it may not always yield optimal decisions for daily HPP operations.

### 3.2 Mathematical Optimization

**FIGURE 7:**
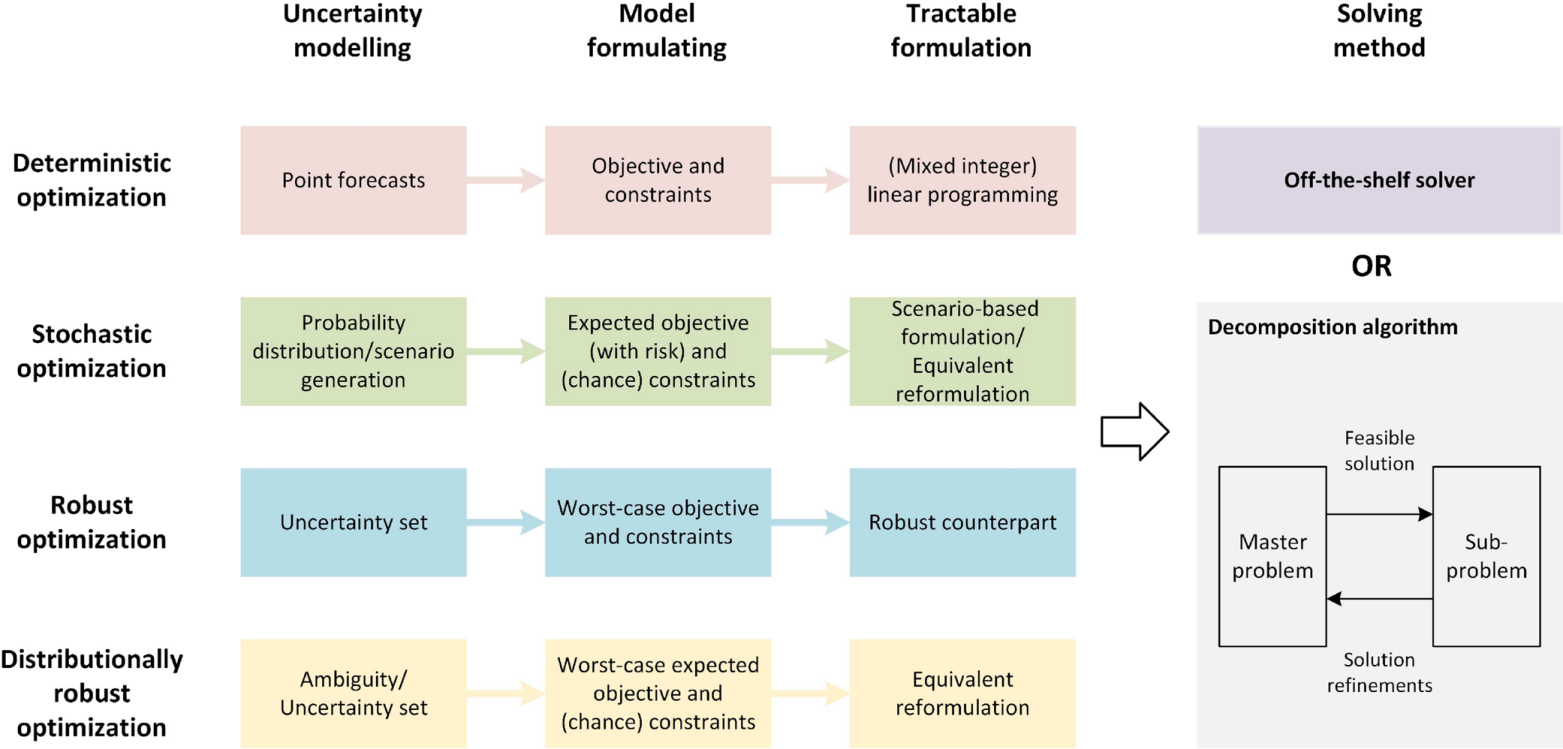
An overview of the process of MO‐based methodology.

Mathematical optimization is a popular choice for modeling EMS because it excels at balancing multiple objectives while adhering to technical and economic constraints. This methodology is favored for its ability to systematically find the best solutions while ensuring efficient and effective uncertainty management. Figure 7 presents the process of implementing MO‐based EMS, including uncertainty modeling, model formulation, tractable reformulation, and problem solving. The detailed information is described in the following subsections.

#### 3.2.1 Deterministic Optimization

DO models incorporate specific forecasts, such as renewable energy output and market prices, into their objective functions and constraints. The general form can be expressed as:

(1) $$\begin{array}{ll} & \max_{x}\;f\left(x,\hat{\xi }\right)\\ & \;s.t.\;g\left(x,\hat{\xi }\right)\leq 0 \end{array}$$

In this formulation, x represents the decision variables, and $\hat{\xi }$ indicates the point forecasts of any uncertain variables. The expressions $f\left(x,\hat{\xi }\right)$ and $g\left(x,\hat{\xi }\right)$ stand for the objective functions and constraints, respectively. The Problem (1) is usually formulated by linear programming or mixed integer linear programming (MILP), as these methods efficiently handle large‐scale optimization problems with linear constraints and objective functions. It allows for the use of powerful off‐the‐shelf commercial solvers, which are well‐developed and capable of efficiently finding the global optimal solution. In the investigation of day‐ahead scheduling for wind‐battery HPPs, Das, Grapperon, et al. (2020) propose an approach enabling participation in spot markets and real‐time balancing. Their framework employs a MILP model, utilizing wind generation forecasts and spot price signals to optimize operational decisions. The MILP is solved by IBM CPLEX implemented by Python. Yang et al. (2018) propose a MILP formulation to optimize the profits of wind‐CSP‐based HPPs. The electric heater is included to generate thermal energy by utilizing excess wind power. The MILP is solved by IBM CPLEX implemented by Matlab. The bidding strategy of hybrid wind‐PV‐hydro plants considering future utilities is studied by Li et al. (2023). The case studies are performed through Matlab. Some studies also explore heuristic approaches, such as particle swarm optimization. For example, An et al. (2019) explore the firmness generation from ydro‐wind‐PV‐battery power plants by minimizing the output power volatility. The problem is solved by the niche particle swarm optimization. While heuristics can offer faster solutions in certain cases, they do not guarantee global optimality and are generally less reliable for complex, large‐scale problems than commercial solvers.

DO‐based EMS is able to provide optimal power schedules. However, it relies on highly accurate forecasts or HPPs possessing substantial dispatchable assets (Kong et al. 2022). Research conducted by Zhu, Lindberg, et al. (2023) indicates that the imperfect forecast of wind power can significantly reduce profits compared to perfect forecasts. The reason is that when available power exceeds forecasts, curtailment may occur due to storage limits, while shortages in available power can lead to market penalties if storage cannotcover the deficit.

#### 3.2.2 Stochastic Optimization

SO is a well‐established technique for formulating EMS models. In this approach, probability distributions are used to represent uncertainties stemming from, for example, renewable power generation. The general mathematical structure is shown below:

(2) $$\begin{array}{ll} & \max_{x}\;E_{P}\left[f\left(x,\tilde{\xi }\right)\right]\\ & \;s.t.\;g\left(x,\tilde{\xi }\right)\leq 0 \end{array}$$

where $\tilde{\xi }$ represents uncertain variables, which follows the probability distribution P. The expectation term $E_{P}\left(\cdot \right)$ in the objective function is usually handled by linear approximation model (Ding et al. 2015) or scenario‐based SO (Tan and Guan 2023; Ming et al. 2018; Gomes et al. 2017; Zhou et al. 2023). In scenario‐based SO, the fundamental idea is to generate a collection of possible realizations for uncertain variables and then optimize the expected objective across these realizations. Numerous scenario‐generation techniques appear in the literature. For instance, Crespo‐Vazquez et al. (2018b) rely on multivariate clustering and recurrent neural networks to create potential energy price and wind power scenarios, while Aghaei et al. (2015) and Akbari et al. (2019) utilize a roulette wheel mechanism to derive their wind power and price scenarios. Meanwhile, Wang et al. (2016) employ conditional kernel density estimation for generating wind forecast scenarios. Since handling a large number of scenarios can become computationally intensive, scenario reduction techniques, such as the simultaneous backward method (Zhan et al. 2020) are often introduced to overcome the issue.

To consider risk measurement, chance‐constraint can be applied on SO, as shown in the Model (3).

(3) $$\begin{array}{ll} & \max_{x}\;E_{P}\left[f\left(x,\tilde{\xi }\right)\right]\\ & \;s.t.\;P\left(g\left(x,\tilde{\xi }\right)\leq 0\right)\geq 1-\epsilon \end{array}$$

Chance constraints ensure probabilistic satisfaction with level 1−ϵ, while allowing the possibility to capture higher benefit opportunities with low risk. Chance constraints often introduce nonlinear and nonconvex conditions into the optimization problem. These conditions can make the problem computationally intractable. They are commonly addressed using reformulation techniques, such as replacing the probabilistic constraints with their deterministic equivalents. Zhao et al. (2019) develop the chance‐constrained SO to ensure power from WPP and CSP follows optimal setpoints with a certain probability. Similarly, Singh and Knueven (2021) use chance constraints to ensure probabilistic satisfaction of promised energy. Chance constraints are applied by Fang and Zhao (2020) to manage uncertainties from wind and solar direct normal irradiance. All the chance constraints in these works use the reformulation technique introduced by Liu et al. (2003) to obtain the deterministic equivalence. The final reformulated models are solved by the commercial solvers.

**FIGURE 8:**
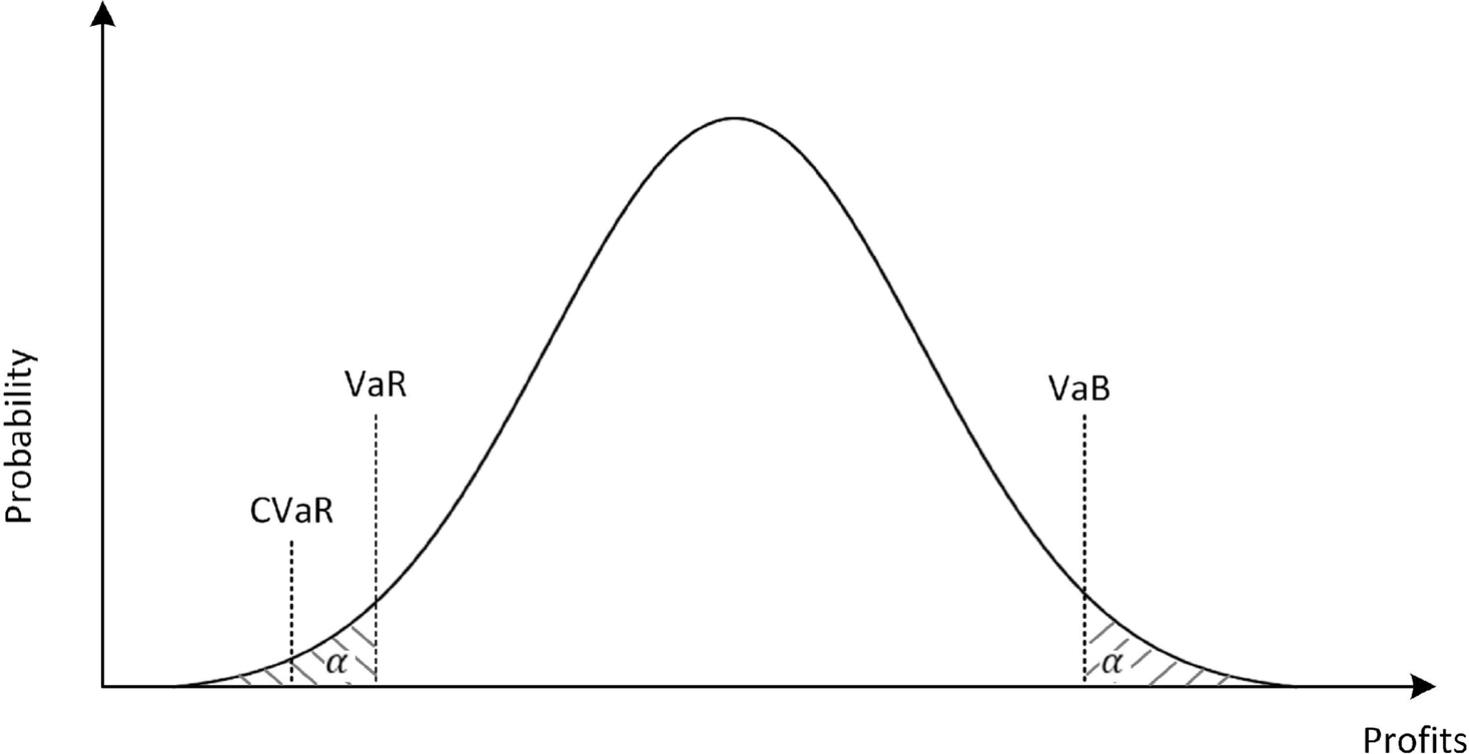
Different risk measurements towards expected profit distribution.

Model (2) is generally classified as a risk‐neutral model. It can be modified to a risk‐averse or risk‐seeking formulation depending on decision‐makers' preferences. Figure 8 demonstrates the expected profit distribution. Most literature studies the risk‐averse formulation, which focuses on the left side of the distribution. Risk‐averse decision‐makers want to avoid those scenarios that cause extremely low profits. A well‐known risk metric is value‐at‐risk (VaR), the minimum profit that the probability of obtaining profits lower than or equal to it is no less than α. To ensure the convexity of the optimization model, the literature usually uses conditional value‐at‐risk (CVaR), which is the expected value of the realized profits below VaR. The general formulation of CVaR‐SO is to maximize the weighted profit expectation and CVaR, as presented in Equation (4).

(4) $$\begin{array}{ll} & \max_{x}\;\left(1-\beta \right)\cdot \sum_{s=1}^{N}\left[p_{s}\cdot f\left(x,{\hat{\xi }}_{s}\right)\right]+\beta \cdot \text{CVaR}\\ & \;s.t.\;g\left(x,{\hat{\xi }}_{s}\right)\leq 0\\ & \;-f\left(x,{\hat{\xi }}_{s}\right)+\gamma -\eta_{s}\leq 0\\ & \;\forall s=1,\cdots ,N \end{array}$$

where the probability of each scenario s is expressed as $p_{s}$. The weight β is introduced to balance objectives and risks. γ represents VaR. $\eta_{s}$ is an auxiliary variable. The formulation of CVaR is shown in Equation (5).

(5) $$\text{CVaR}=\gamma -\frac{1}{1-\alpha }\sum_{s=1}^{N}p_{s}\cdot \eta_{s}$$

The CVaR‐based SO is applied by Khaloie et al. (2020, 2022) to model optimal market participation of HPPs with different configurations. They look into many kinds of uncertainty sources from different renewable generation and market prices. A similar implementation is also done by Nieta et al. (2016), where apart from wind power and marker price, scenarios of water inflows are also considered in their model. Decision‐makers may also be interested in the right side of expected profit distribution, where HPPs could achieve substantial windfall profits in certain extreme scenarios. To capture it, Xiao et al. (2024) introduce the value‐at‐best (VaB), which is the maximum profit that the probability of obtaining profits higher than or equal to it is no less than α. Then, a more generalized SO formulation is proposed through a weighted average of the expected profits, VaB, and CVaR, as presented in Equation (6). The risk preference can be adjusted by tuning the weights.

(6) $$\;\max_{x}\;\left(1-\beta_{1}-\beta_{2}\right)\cdot \sum_{s=1}^{N}\left[p_{s}\cdot f\left(x,{\hat{\xi }}_{s}\right)\right]+\beta_{1}\cdot \text{CVaR}+\beta_{2}\cdot \mathrm{VaB}$$

It is noted that both Equations (4) and (6) are already the scenario‐based formulation, which can be directly solved by the solvers.

#### 3.2.3 Robust Optimization

Unlike SO, which focuses on optimizing the expected objective values, RO optimizes the worst‐case objective function. The formulation is expressed as:

(7) $$\begin{array}{ll} & \max_{x}\underset{\tilde{\xi }\in \Omega }{\inf}\;f\left(x,\tilde{\xi }\right)\\ & \;s.t.\;g\left(x,\tilde{\xi }\right)\leq 0 \end{array}$$

where Ω represents the uncertainty set encompassing all possible values of $\tilde{\xi }$. The basic RO model can be enhanced with CVaR (Wang et al. 2019) and chance constraints (Tan et al. 2023). A key benefit of RO is its independence from probability distribution (Ben‐Tal et al. 2009). The RO formulation is usually a bi‐level optimization. The robust counterpart is usually obtained based on the inner optimization's Karush–Kuhn–Tucker conditions to handle the complexity. The robust counterpart is usually solved by off‐the‐shelf solvers. Alternatively, the bilevel formulation can also be decomposed into a master problem and a subproblem, which are solved iteratively to get the optimal solution, as shown in Figure 7.

To handle uncertainties arising from the price of intraday demand response exchange markets, the load of demand response providers, and the thermal output of CSP solar fields, Khaloie, Anvari‐Moghaddam, Contreras, and Siano (2021) propose an interval‐based robust optimization strategy. Their framework simultaneously maximizes the midpoint between the worst‐ and best‐case profits and minimizes the gap between these extremes, thereby enabling robust and risk‐averse operational decisions.

In a different study, Attarha, Amjady, Dehghan, and Vatani (2018) adopt robust optimization with a polyhedral uncertainty set to address variations in wind power generation and day‐ahead market prices. By utilizing a tri‐level robust operation model, they demonstrate that co‐locating wind plants with compressed air energy storage achieves superior economic performance compared to operating each asset in isolation. Building on similar principles, Attarha, Amjady, and Dehghan (2018) incorporate affine decision rules into robust optimization to account for fluctuations in solar output and market prices. This approach eliminates the need for decomposition techniques and reveals that a solar power plant co‐located with battery storage is more profitable than separate operations.

In another work, Mohamed et al. (2020) develop a robust model focused on day‐ahead scheduling of wind‐battery hybrid plants. Their approach, which treats the HPP as a price maker and includes transmission‐switching constraints, effectively reduces congestion, increases wind utilization, and enhances overall revenue. Lastly, Pousinho et al. (2015) evaluate robust operational decisions for hybrid CSP‐fossil power plants under bilateral contracts in day‐ahead markets, applying a polyhedral uncertainty set to address both solar thermal power and market price uncertainties.

#### 3.2.4 Distributionally Robust Optimization

DRO is a widely adopted method for tackling uncertainty, where all plausible probability distributions, derived from the decision maker's prior knowledge, are grouped into an ambiguity set P. The optimal decision is subsequently determined by considering the most adverse distribution within this set (Wiesemann et al. 2014). The formulation can be expressed as follows:

(8) $$\begin{array}{ll} & \max_{x}\underset{P\in P}{\inf}\;E_{P}\left[f\left(x,\tilde{\xi }\right)\right]\\ & \;s.t.\;g\left(x,\tilde{\xi }\right)\leq 0 \end{array}$$

In this formulation, P is the probability distribution, which resides in the specified ambiguity set. One notable strength of DRO lies in its fusion of features from SO and RO. Unlike SO, it does not rely on a fixed probability distribution, yet it still retains the probabilistic information that RO typically discards (Zhu et al. 2019). However, apart from the bilevel formulation, the probability distribution is also a decision variable, making model complexity even higher than RO. As Figure 7 demonstrates, the first step to implement DRO is constructing ambiguity sets. This is usually done with moment information or the Wasserstein metric in the literature.

Han and Hug (2020) utilize DRO to model bidding strategies for wind‐battery HPPs in day‐ahead markets. The HPP is considered a price maker. The first‐order moment‐based ambiguity set models the uncertainty of wind power forecasting errors. However, the recourse function is simplified by affine functions, which reduce the model's complexity and approximate how the plant responds to realized forecasting errors. Huang et al. (2020) study the EMS of wind‐battery HPPs at a portfolio level. They further include the second‐order moment information in the ambiguity set to model wind power uncertainty and market price forecasts. This narrows down the ambiguity set and reduces the conservativeness. To verify the proposed model's performance, real‐world ERCOT market prices and NREL wind datasets are employed in the evaluation. In related work, Xu et al. (2020) study day‐ahead scheduling for wind‐battery HPPs using a moment‐based DRO framework. Their model also addresses grid interconnection constraints via a distributionally robust chance constraint (DRCC), illustrated in Equation (9). Under the worst‐case distribution, the DRCC enforces a specified probability of constraint satisfaction, thereby incorporating risk into the decision‐making process. Simulation results indicate that the proposed model consistently adheres to the grid connection requirements.

(9) $$\begin{array}{ll} & \max_{x}\underset{P\in P}{\inf}\;E_{P}\left[f\left(x,\tilde{\xi }\right)\right]\\ & \;s.t.\;\underset{P\in P}{\inf}P\left(g\left(x,\tilde{\xi }\right)\leq 0\right)\geq 1-\epsilon \end{array}$$

Ma, Hu, and Song (2022) apply a Wasserstein metric‐based DRO combined with DRCC for a wind‐battery plant to provide frequency and automatic generation control services. DRCCs are used to model uncertainties in ancillary service requirements uncertainties.

With most DRO studies focusing on profit maximization, in many cases, a limited degree of constraint violation under extreme circumstances can be acceptable if the related penalties are outweighed by greater profitability. Thus, operators must balance the potential gains from market participation against the risk of incurring violations.

### 3.3 Model Predictive Control

MPC uses a finite prediction horizon to optimize upcoming control actions. The formulation can be expressed as:

(10) $$\begin{array}{ll} & \min_{x}\;f\left(x\right)\\ & \;s.t.\;s\left(t+1\right)=A\cdot s\left(t\right)+B\cdot x\left(t\right)\\ & \;y\left(k\right)=C\cdot s\left(t\right) \end{array}$$

**FIGURE 9:**
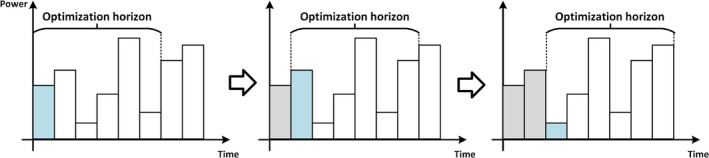
Illustration of the receding horizon policy of MPC.

Within this formulation, $x\left(t\right),s\left(t\right),y\left(t\right)$ denote the control, state, and output variables at discrete time step t. The constraints encode the underlying system dynamics in discrete time. A defining aspect of MPC is its receding horizon principle (Mattingley et al. 2011). As illustrated in Figure 9, only the control action for the current step is actually applied, and then the time window advances by one step to run the optimization again. This iterative mechanism naturally implements the concept of rolling optimization.

From the market participation perspective, MPC is well‐suited for scenarios requiring continuous power schedule change with frequent power and price forecast updates. The objective function is usually to maximize profits of future N steps, as demonstrated in Equation (11)

(11) $$\max\sum_{t=0}^{N}\left(\lambda_{t}P_{t}-C_{t}\right)$$

For example, Khalid et al. (2018) examine the optimal operation of an HPP in the Australian energy market, which clears the market every 5 minutes. The study uses an MPC model to determine the optimal power output of the HPP, aiming to maximize market revenues over the next six dispatch intervals (30 min). However, only the optimal schedule for the first 5‐minute interval is submitted to the market. Then, the model is repeated at the next interval with updated wind power and electricity price forecasts. Likewise, Abdeltawab and Mohamed (2015) introduced a market‐oriented EMS tailored to wind‐battery HPPs operating in Alberta's electricity market. Their method aims to maximize profit while extending battery lifespan, achieved through an MPC‐driven constraint optimizer that uses a daily time horizon and updates hourly. Meanwhile, Taha et al. (2018) proposed an online MPC‐EMS for HPPs in the same region. The objective is to minimize operation costs and pollution gas emissions. It is noted that in markets where decisions are made once and not revisited, such as the day‐ahead market, MPC does not differ significantly from the deterministic optimization discussed in Section 3.2.1. Additionally, MPC is also used to manage energy in non‐market‐oriented scenarios. For example, Dieulot et al. (2015) apply MPC to oversee short‐term operations in a hybrid system comprising solar photovoltaics, battery storage, and a gas microturbine. Their approach aims to minimize overall costs, which include gas consumption, carbon dioxide emission charges, battery cycling expenses, and various tariff‐related fees, all within a receding horizon framework.

### 3.4 Deep Reinforcement Learning

DRL utilizes the powerful function‐approximation capabilities of deep neural networks with the adaptive decision‐making processes of reinforcement learning. Recently, DRL has gained considerable attention in the smart grid domain by tackling highly complex challenges such as microgrid energy coordination (Muriithi and Chowdhury 2021; Kolodziejczyk et al. 2021) and devising optimal bidding strategies for energy storage (Cao et al. 2020; Dong et al. 2021). Within the context of HPP energy management, DRL techniques have been employed in designing multi‐timescale bidding strategies for both day‐ahead and real‐time markets (Ochoa et al. 2022) and for photovoltaic battery capacity scheduling (Huang and Wang 2020).

**FIGURE 10:**
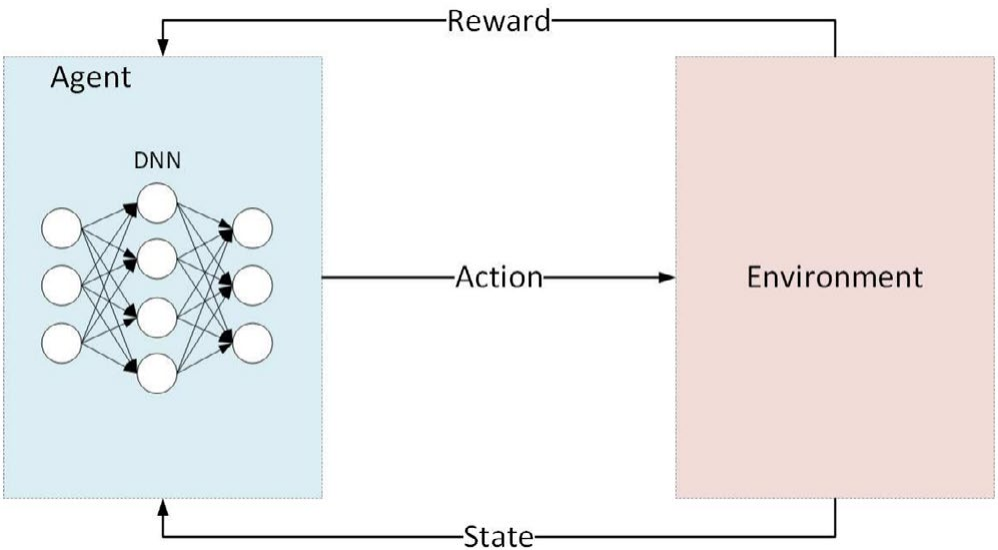
Framework of DRL.

A typical DRL framework features an agent that observes the environment, selects actions, and receives rewards, as illustrated in Figure 10. This relationship between the agent and the environment is frequently modeled as a markov decision process (MDP) (Otterlo and Wiering 2012), wherein the fundamental ingredients are the state space, action space, and reward function. As an example, Huang and Wang (2020) apply DRL to schedule the capacity of HPPs integrating PV and BESS, participating in both energy and frequency regulation markets. Their approach encompasses three operating modes for the PV‐BESS‐based HPP: selling PV output directly in the energy market, leveraging battery storage for energy arbitrage, and providing regulation services. The corresponding MDP elements in their study are formulated as follows:

- State space: the state $s_{t}$ includes the battery state of charge, market prices, and available PV power, and so on.
- Action space: the action $a_{t}$ corresponds to a series of capacity dispatch decisions on the stacked services, that is, directly sold in energy markets, energy arbitrage, and regulation service.
- Reward function: the reward function $r_{t}=B_{t}^{\mathrm{EA}}+B_{t}^{\mathrm{PV}}+B_{t}^{f}-C_{t}$ is defined as net profit of the HPP during hour t, equaling to the revenues from the stacked services minus the battery degradation costs $C_{t}$.

To solve the above DRL model, it is important to find the mapping between actions and status, which is approximated by the deep neural network. The proximal policy optimization is applied to solve the DRL model.

While many DRL applications leverage a MDP formulation, it is not strictly required. For instance, Ochoa et al. (2022) develop a multi‐agent DRL framework for PV‐battery hybrid plants taking part in day‐ahead and real‐time electricity markets. Their setup features two agents employing multi‐view neural networks with recurrent layers to convert observed system data into optimal bidding strategies, all without explicitly defining the problem as an MDP.

### 3.5 Stochastic Dynamic Programming Based EMS

**FIGURE 11:**
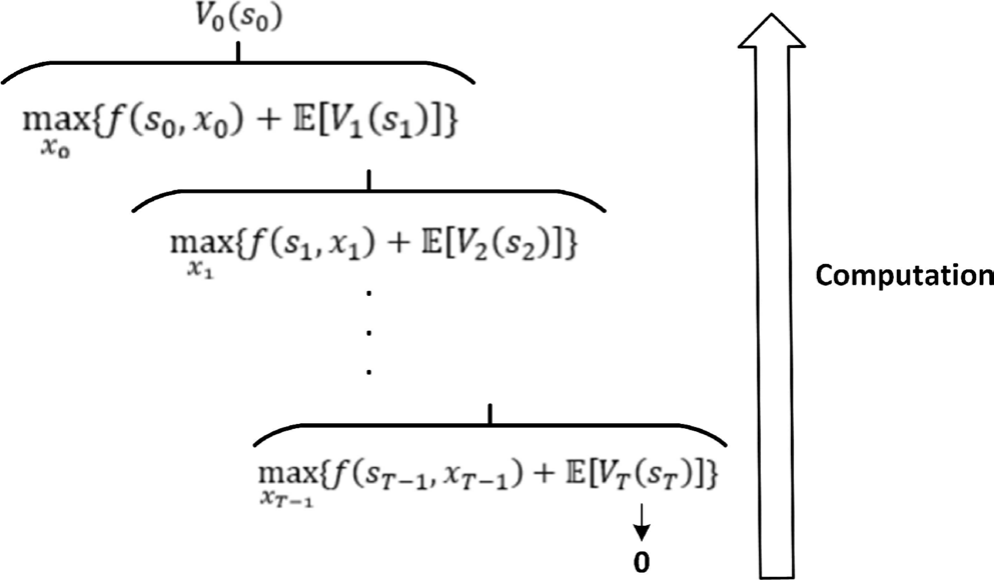
Illustration of the backward recursion method to solve SDP.

SDP builds on conventional dynamic programming and has been employed by Loukatou et al. (2021) and Keles and Dehler‐Holland (2022) to assess the economic prospects of wind‐storage and solar‐storage HPPs, respectively. Implementing SDP necessitates expressing the underlying optimization problem in terms of the Bellman equation (Bellman 1966), often formulated as follows:

(12) $$V_{t}\left(s_{t}\right)=\max_{x_{t}}\;\left\{f\left(s_{t},x_{t}\right)+E\left[V_{t+1}\left(s_{t+1}\right)\right]\right\}$$

where $V_{t}\left(s_{t}\right)$ is the accumulated profits from period t to T with the state $s_{t}$. The state $s_{t}$ usually represents the stochastic renewable power and market prices, as well as the state of charge of the storage at the beginning of the current time step t. $f\left(s_{t},x_{t}\right)$ is the profit at period t. $x_{t}$ represents decision variables, which are the charging and discharging power of storage. The backward recursion method is usually applied to solve the SDP formulated in Equation (12). As demonstrated in Figure 11, the computation starts from the end time T, where the profit is assumed to be 0. This is the terminal condition, setting the basis for backward recursion. Beginning at this terminal state, the SDP process calculates the accumulated profits backward, moving from time T−1 to the initial time period. In the SDP process, optimization occurs at every step of the backward recursion for each state and time, making it computationally intensive.

### 3.6 Comparison of EMS Methodologies

Based on the previous discussion, the overview of the strengths and weaknesses of all reviewed methods is summarized in Table 2. Although many methods have been applied to model the EMS of HPPs, comparing them is challenging due to the fact that methods are being tested on distinctive HPP configurations, as summarized in Section 2. However, a few studies have undertaken comparative analyses within the same configuration, providing valuable insights into the relative performance of different EMS approaches. Zhu et al. (2024) compare DRO‐based EMS with the DO and SO‐based EMSs for hybrid wind‐battery power plants in Nordic day‐ahead markets considering imbalance settlement. By adjusting the parameters of the DRO, the economic performance ranks highest with DRO, followed by risk‐neutral SO and then DO. DRO can make more robust offering strategies, especially in the market environment, which has higher penalties for imbalanced energy. Similarly, Han and Hug (2020) conduct a one‐year simulation on hybrid wind‐battery plants using Nord Pool data, showing that the DRO model can achieve higher revenues than the bidding with deterministic forecasts. Ochoa et al. (2022) compare the DRL with SO and RO for the PV‐battery HPP using the US market data. The results indicate that DRL has better economic performance than SO and RO. Besides, DRL also offers a significant advantage in computational time compared with SO and RO.

**TABLE 2:** Strengths and weaknesses of the EMS methodologies.

	Strengths	Weaknesses
Rule‐based	Fast computational time Simple to implement and understand	Limited flexibility and adaptability May not handle uncertainties well
Deterministic optimization	Easier to formulate and solve	Does not account for uncertainties
Stochastic optimization	Explicitly incorporates uncertainties Scalable to different risk preferences	Computational complexity due to the duplication of constraints across a large number of scenarios Rely on probability distribution information
Robust optimization	Do not rely on exact probability distribution information Ensures performance under the worst‐case scenario	Solutions tend to be conservative
Distributionally Robust optimization	Trade‐off between robustness and economy	Rely on complicated reformulation techniques or decomposition algorithms
Model predictive control	Suitable to problems with continuous decision updates with new data	High computational demand
Deep reinforcement learning	Learns optimal strategies from data Handles complex and high‐dimensional problems	Requires large amounts of training data Solutions may not be feasible
Stochastic dynamic programming	Consider future potential benefits	Complex implementation High computational demand

In summary, MO remains the mainstream methodology for EMS in HPPs due to its established frameworks, robustness, and proven performance in various applications. DRO, as a traditional MO‐based methodology, demonstrates commendable results. DRL emerges as a highly promising methodology for EMS in HPPs, particularly due to its strong performance in maximizing profits. However, the effectiveness of DRL is contingent upon the availability of substantial data and the considerable effort required for model training. Additionally, the complexity of DRL models necessitates advanced computational resources and expertise. There is a noticeable gap in the literature regarding direct comparisons between DRO and DRL under identical case scenarios, making it difficult to ascertain their relative advantages in practical applications. Rule‐based methodologies, on the other hand, offer the advantage of fast computational times, making them suitable for real‐time applications. However, these methodologies are highly case‐dependent, meaning their effectiveness can vary significantly depending on the specific conditions and rules applied. This case dependency limits their generalizability and adaptability across different scenarios. MPC and SDP, while powerful in certain contexts, are not considered mainstream methods for EMS in HPPs due to their specific features.

## 4 EMS for Market Alignment and System Needs

Electricity markets vary widely by region and country, depending on their level of development, resource availability, institutional arrangements, and policy objectives. In Europe, the electricity market is largely deregulated and liberalized. The electricity market is divided into several regional markets interconnected by cross‐border transmission lines. Different regions and countries have similar market designs. The European Network of Transmission System Operators for Electricity coordinates the operation and planning of the transmission system across Europe. In North America, the electricity market is also divided into several regions, but they are largely isolated from each other due to limited interconnections. The regions are organized as regional transmission organizations (RTOs) or independent system operators (ISOs), which operate wholesale markets and manage the transmission grid, or as non‐market areas, where utilities or other entities are responsible for these functions. The Australian National Electricity Market operates as a wholesale market where electricity generators, retailers, and large consumers participate. It covers Australia's eastern and southeastern states and the Australian Capital Territory.

**FIGURE 12:**
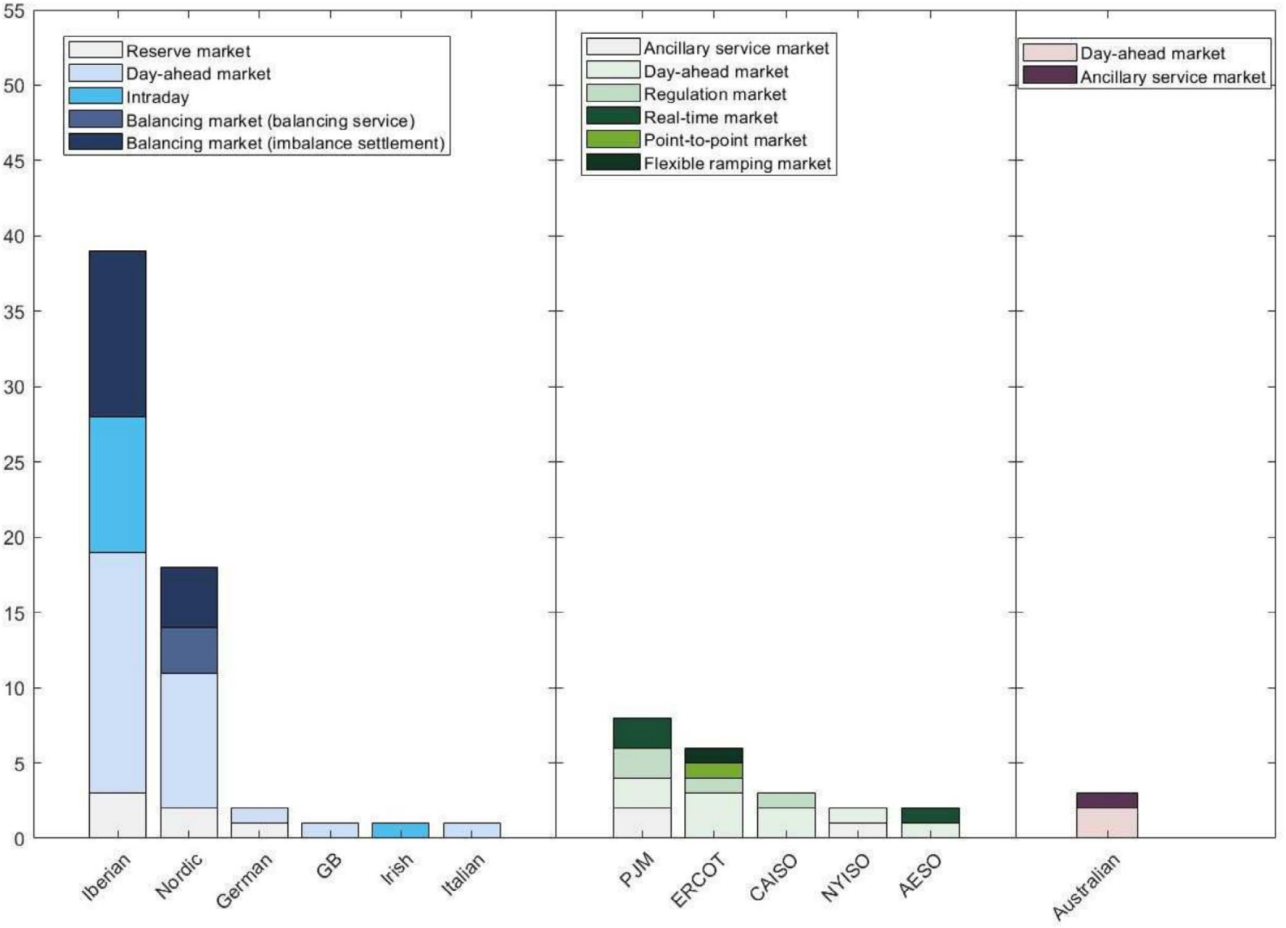
Classification of the literature based on EMS considering electricity markets in different regions and countries.

This section addresses HPPs' participation in electricity markets by classifying and discussing the literature targeting different regions and countries. Figure 12 demonstrates the considered markets in different regions and countries. It is observed that a majority of studies on the EMS of HPPs focus on the European electricity markets, with a lesser extent of research dedicated to the North American electricity markets and only minimal attention to the Australian context.

### 4.1 The European Electricity Markets

**FIGURE 13:**
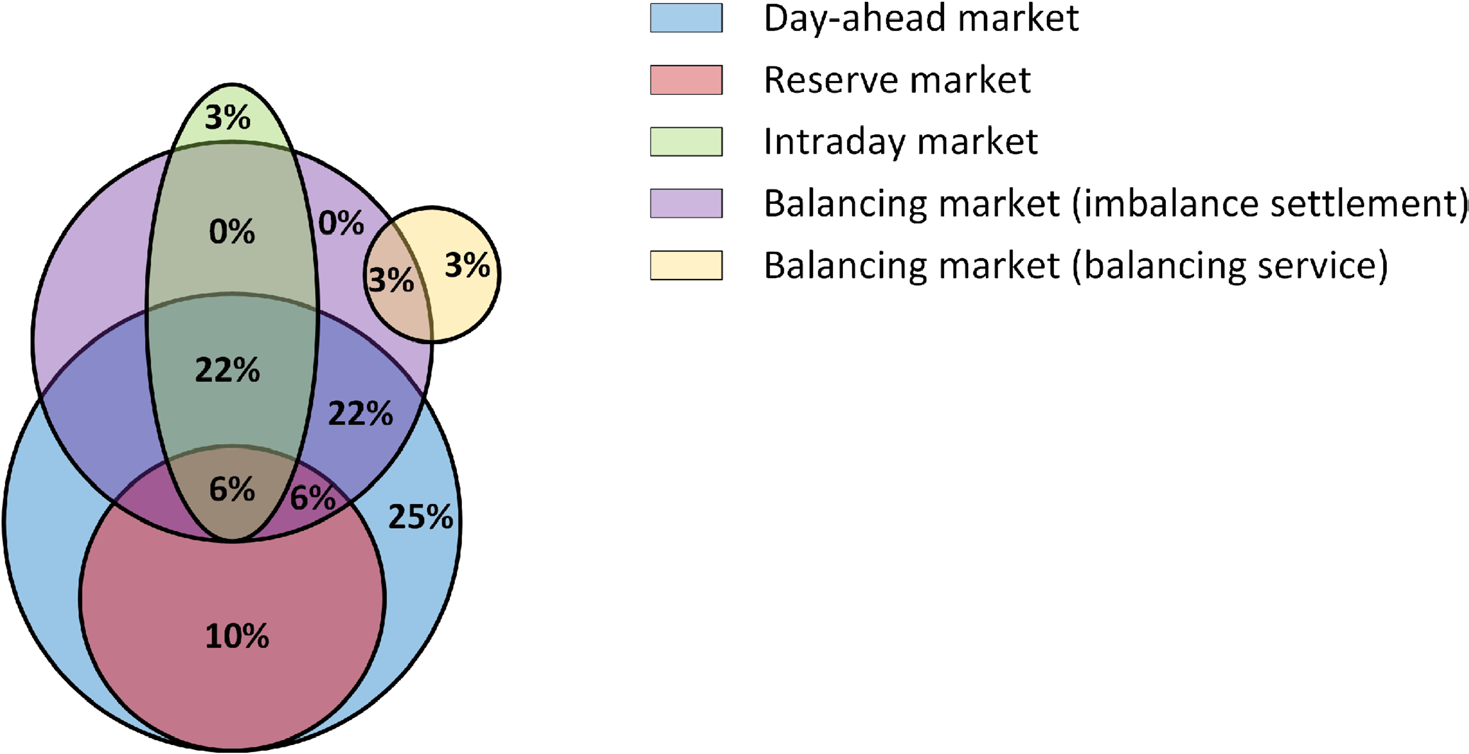
Venn diagram of European electricity markets considered in the EMS.

The European electricity markets can be classified as day‐ahead reserve markets, day‐ahead spot markets, intraday markets, and balancing markets. Figure 13 demonstrates the statistics of the percentage of papers that study HPPs in these markets. It is noted that balancing markets assume two roles: the trade of balancing services and the settlement of imbalances, which are separated in Figure 13.

The energy committed in day‐ahead spot markets serves as a reference during the settlement of HPPs' generation. Therefore, the literature extensively explores the HPPs' participation in day‐ahead spot markets. Twenty‐five percent of the studies consider HPPs' participation in solely spot markets (Ding et al. 2017; Darvishi et al. 2020; Lindberg, Zhu, et al. 2023; Gomes et al. 2023), while 22% of the studies consider the spot markets with anticipating minimum imbalance costs in balancing markets (Silva et al. 2022; Ding et al. 2016). Moreover, the day‐ahead participation of HPPs in reserve and spot markets is always jointly optimized. For instance, Crespo‐Vazquez et al. (2018a) study day‐ahead optimization for hybrid wind‐storage plants in reserve and spot markets. The optimal bidding of compressed air energy storage with wind and thermal generation units in reserve and spot markets are studied by Akbari et al. (2019). It is noted that different products can traded in the reserve markets. For instance, the potential of providing frequency containment normal reserve from HPPs is studied by (Zhan et al. 2020). Furthermore, Intraday markets that open for re‐offers and bids due to foreseen factors, for example, day‐ahead and hour‐ahead forecasting errors (Lawati et al. 2021), are also studied in the literature. The optimal participation of HPPs in bilateral contract‐based intraday markets is studied by (Ghavidel et al. 2019). Notably, these intraday markets are frequently jointly optimized with spot and balancing markets. On the other hand, the optimal participation of HPPs in Irish auction‐based intraday markets is studied by (Bakhtvar and Al‐Hinai 2021). Finally, the HPPs' optimal engagement in hour‐ahead balancing markets to offer essential balancing services is conducted in (Zhu, Das, et al. 2023).

Although there have been many works on EU markets, a significant body of research suggests that HPPs are temporally economically infeasible. Loukatou et al. (2021) compare the value of HPPs in GB wholesale markets with power purchase agreements. Case studies indicate that, even with subsidies, the profits can be unfavorable. When power purchase agreements are used, a wind power plant attains a higher net present value compared to an HPP operating in the wholesale market. However, once subsidies are removed, both scenarios exhibit negative net present values, suggesting that market participation may not be advisable for HPPs. A similar observation is reported by Keles and Dehler‐Holland (2022) for photovoltaic‐storage HPPs in the German market, using 2015 data. Their findings suggest that exclusive participation in Germany's spot and reserve markets is currently not economically viable for PV‐storage systems. The primary factor contributing to the economic challenges of HPPs is the high cost associated with batteries. Additionally, Klyve et al. (2023) study the value of HPPs in Nordic manual frequency restoration reserve markets. The results indicate that using batteries to provide manual frequency restoration reserve is more profitable than eliminating forecasting errors. In conclusion, battery prices are anticipated to decrease in the coming years. Besides, with the growing integration of renewable energy into electricity markets, future electricity prices are expected to become more volatile, leading to greater balancing requirements in power systems. Therefore, it is optimistic that HPPs will be profitable in the future. Further studies are needed to include a broader range of markets and thoroughly investigate the economic viability of HPPs in European markets.

### 4.2 The North American Electricity Markets

In existing studies, considerable research attention is devoted to exploring the optimal participation of HPPs in the electricity markets of five distinct ISOs or RTOs. They are PJM, Electric Reliability Council of Texas (ERCOT), California ISO (CAISO), New York ISO (NYISO), and Alberta Electric System Operator (AESO). The research predominantly emphasizes the technical effectiveness of HPPs, aiming to reduce generation uncertainty and provide frequency support within the associated electricity markets.

Taha et al. (2018) propose an online EMS to help HPPs reduce the costs of the grid to compensate for forecasting errors under the one‐spike and two‐spike price profile in Alberta electricity markets. In PJM, there is a traditional regulation signal (RegA) that is designed to balance the grid by matching generation with load to manage frequency deviations. Resources providing RegA are capable of both increasing and decreasing output or consumption symmetrically in response to the grid operator's commands. The dynamic regulation signal (RegD) is an alternative regulation service that allows for faster, more flexible response rates (every 2 or 4 s) compared to RegA. This service is particularly well suited for newer technologies like batteries, which can change their output very rapidly. He et al. (2016) introduce a collaborative strategy linking wind power and battery storage to fulfill real‐time RegD obligations in tandem with their day‐ahead regulation commitments. Their approach prioritizes wind energy in tracking the regulation signal, with the storage system stepping in to address deficits and correct inaccuracies. This setup not only capitalizes on the precise ramping ability of batteries but also preserves battery health by limiting excessive cycling. Similarly, Huang and Wang (2020) present a deep reinforcement learning‐based approach that can effectively coordinate the PV‐battery system to track the RegD signal for frequency regulation service. Besides, flexible ramping markets provide additional ramping resources to manage unexpected changes in system load or renewable energy output, ensuring the grid can handle short‐term uncertainties not captured in day‐ahead or real‐time forecasts. Unlike regulation markets that manage second‐to‐second fluctuations to maintain a stable frequency, flexible ramping markets address forecast inaccuracies and variability within hours. Huang et al. (2020) provide a practical example by examining how hybrid wind‐storage plants participate in flexible ramping markets within ERCOT. Their simulations illustrate that such HPPs effectively enhance system flexibility, contribute to new revenue streams, and decrease portfolio risks, underscoring their value in flexible ramping markets.

### 4.3 The Australian Electricity Markets

Australia has completely different market designs from those of the EU and North America. It consists of only energy markets and frequency control ancillary service markets, where the dispatch and offering/bidding of generators and demand are all within 5‐min resolutions. To cope with such frequent trades, a receding horizon approach is proposed by Khalid et al. (2018) for hybrid wind‐battery plants to participate in energy markets. According to their simulation results, a 50 AUD/MWh subsidy is required to motivate the installation of energy storage along with the wind power plant. Moolman et al. (2021) also observe similar results that HPPs participating in energy markets only are economically infeasible due to the high costs of batteries. Besides, an analysis by Naemi et al. (2022) reveals that participating in both energy and ancillary service markets brings greater returns to hybrid wind‐battery plants than participating only in energy markets. These analyses highlight the importance of considering government interventions and industry innovations to help mitigate the high costs associated with battery storage, which present significant challenges to the economic viability of HPPs in Australia.

As discussed above, EMS plays a critical role in optimizing the operation of HPPs to maximize potential profits in various electricity markets. Beyond economic considerations, EMS also supports the technical integration of HPPs into the power system by addressing specific system needs, such as firm power provision. For instance, IEA PVPS Task 16 (2023) demonstrates the feasibility of co‐locating PV systems with batteries to deliver firm power through proactive curtailment strategies. In practice, EMS is essential for determining optimal proactive curtailment strategies, enabling HPPs to balance renewable generation with storage and ensure reliable power delivery. However, Pierro et al. (2020) identify a paradox in the Italian context, where the current market mechanism based on a single‐price imbalance settlement scheme fails to adequately incentivize HPPs to provide firm power. Similarly, Clò and Fumagalli (2019) highlight that dual‐price imbalance settlement schemes are more effective in facilitating firm power provision by power plants as compared to single‐price schemes. Despite these observations, many EU countries adopt single‐price settlement mechanisms for the imbalance between actual generation and scheduled generation of HPPs (eSett 2023). This underscores the need for market reforms that align incentives with the technical capabilities of HPPs, ensuring their potential for delivering firm power is fully realized.

## 5 Uncertainties Considered in EMS

**FIGURE 14:**
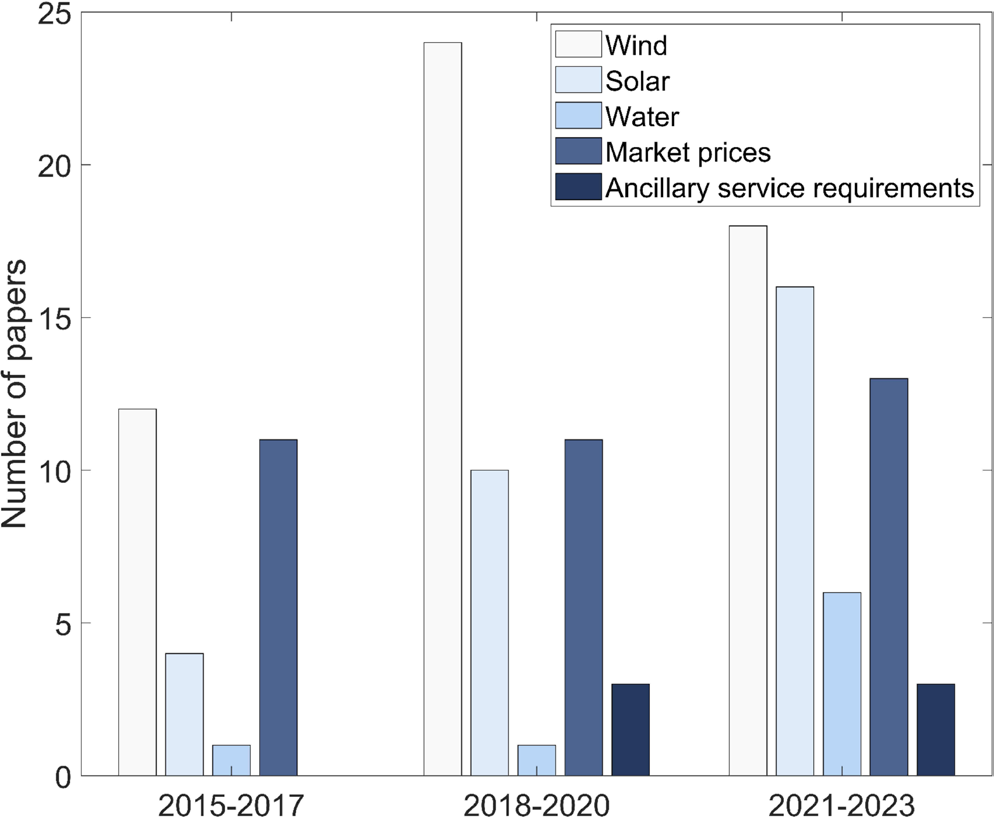
Uncertainties considered in EMS.

Integrating renewable energy sources and allowing HPPs to participate in various electricity markets has led to increased uncertainties, posing significant challenges to their economic operations. These uncertainties make it challenging to establish perfect generation schedules. Figure 14 summarizes the identified uncertainty sources based on the reviewed papers from 2015 to 2023. As shown, the key uncertainty sources can be grouped into three categories: renewable energy uncertainty, market price uncertainty, and uncertainty in ancillary service demands.

### 5.1 Renewable Source

The uncertainty associated with renewable energy sources in HPPs primarily stems from the inherent variability of resources like wind speeds and solar irradiation (Abhinav and Pindoriya 2021). For hydropower plants within HPPs, this uncertainty is further compounded by fluctuations in water inflows (Ming et al. 2019), which depend on variables such as weather conditions and upstream water availability. The HPP generation based on these renewable energy sources remains unpredictable until real‐time operation begins. Accurately predicting renewable generation is inherently difficult, as it depends on volatile environmental conditions such as weather patterns and topographic features. Consequently, basing HPP operations solely on forecasts may not deliver adequate precision, highlighting the necessity for advanced energy management strategies that can accommodate uncertainties. It is notable that all reviewed studies addressing uncertainties in HPP modeling include at least one renewable energy‐related uncertainty.

### 5.2 Market Price

Price uncertainties in HPP operations span multiple markets, such as spot markets, capacity markets, and energy activation markets. Because bids must be submitted ahead of actual price realization, reliable forecasting becomes indispensable for scheduling and dispatch. Yet, forecasting accuracy can be undermined by diverse factors, including transmission constraints and the tendency for market prices to exhibit inverse relationships with renewable output (Hu et al. 2010; Martinez‐Anido et al. 2016), and so on. Although a study from Zhu, Lindberg, et al. (2023) shows that spot price uncertainty does not have significant impacts on HPP profits, it is still important to have accurate forecasts. Balancing prices, in particular, can be highly uncertain, as they depend on whether the power system is experiencing generation surpluses or deficits. Occasional events like network outages may cause price spikes (Bottieau et al. 2022), further increasing uncertainty. Given these considerations, accurately predicting market prices remains a challenge, underscoring the importance of effective uncertainty management strategies for HPP operators aiming to maximize profits in electricity markets.

### 5.3 Ancillary Service Requirements

Incorporating energy storage into HPPs makes it feasible for them to deliver ancillary services. Under typical market rules, HPPs must reserve a certain power capacity on a day‐ahead basis. However, the exact specifications for ancillary services are often determined closer to real time, creating uncertainty in capacity scheduling. Recognizing this, several studies (Akbari et al. 2019; Ma, Hu, and Song 2022) treat ancillary service requirements as uncertain variables in their modeling approaches. By explicitly accounting for this unpredictability, HPPs can more effectively allocate power and enhance the reliability of the ancillary services they offer.

### 5.4 Others

Beyond these principal sources, some studies account for a variety of additional uncertainties. For instance, congestion of transmission lines (Mohamed et al. 2020), demand response (Ghalelou et al. 2016), and temperature (Ghalelou et al. 2016) have all been incorporated into models aimed at more accurately capturing the real‐world complexities faced by HPP operators.

Addressing the operational uncertainties in HPPs has prompted various studies aimed at enhancing forecasting accuracy, such as using physics‐informed machine learning (Pombo et al. 2022) or random forest techniques (Lindberg, Lingfors, et al. 2023). Beyond forecasting, numerous works employ discrete scenarios to approximate continuous probability distributions. For example, wind power and ancillary service requirement scenarios are generated by the k‐means algorithm by Crespo‐Vazquez et al. (2018a). Li et al. (2020) review more scenario generation algorithms.

In some cases, probabilities are not used at all; for example, Khaloie, Anvari‐Moghaddam, Hatziargyriou, and Contreras (2021) represent the uncertainty of demand response prices through interval bounds that capture all potential outcomes without relying on specific probability distributions. Typically, HPPs must consider multiple sources of uncertainty such as renewable output and market prices, which may be correlated. Capturing these correlations is crucial for accurately reflecting uncertainty impacts on operational and economic decisions. Furthermore, each source of uncertainty can show autocorrelation over time. An illustrative example comes from the Grand Maison wind farm in France, where wind speed forecast errors were found to be positively autocorrelated (Haessig et al. 2015). Recognizing both correlations among different uncertainties and autocorrelation within each uncertainty factor is therefore an essential consideration for more robust HPP scheduling and operation.

## 6 Industrial EMS Tools

In addition to the diverse EMS approaches stemming from academic research, the tangible advantages of HPPs have motivated the industrial sector to develop specialized EMS solutions. Table 3 summarizes industrial EMS tools and their applications in real‐world HPPs. However, all the EMS tools for HPPs are proprietary in the industry. FLEXIQ Dispatcher and GEMS IntelliBidder are discussed in this section based on public knowledge.

**TABLE 3:** Overview of industrial EMS tools and real‐world applications.

Reference	Tool	Developer	Application and location
GE Renewable Energy 2021	FLEXIQ Dispatcher	General Electric	Lenox Solar/Energy Storage, USA
(Wärtsilä, 2022b)	GEMS IntelliBidder	Wärtsilä	Hickory Park, USA
Petersen et al. 2018	VestasOnline Power Plant Controller	Vestas	Kennedy Energy Park, Australia; Terna Energy Park Power Plant, Greece; Lem Kær HPP, Denmark
Pombo et al. 2021	Hybrid Power Plant Controller	Vattenfall	Haringvliet HPP, Netherland

General electric renewable energy has developed FLEXIQ, a comprehensive digital platform aimed at optimizing HPPs (GE Renewable Energy 2021). One of its key components, FLEXIQ Dispatcher, functions as a high‐level controller built using MPC optimization. FLEXIQ Dispatcher gathers information from an HPP supervisory control and data acquisition system, integrates forecasts of renewable power and market prices, and then generates at most 48‐h‐ahead battery operation schedules. This schedule is continuously refined as real‐time operations approach and forecast accuracy improves. The FLEXIQ Dispatcher has many functions, including renewable power smoothing, battery lifetime management, and load‐following, and so on. According to DNV's performance test, the FLEXIQ Dispatcher demonstrates benefits over rule‐based dispatch strategies and nodal price optimization approaches General Electric Company 2021. More specifically, the test demonstrates that the FLEXIQ Dispatcher surpasses a conventional industry‐standard approach in several key performance areas, including minimizing ramping rate violations, more effectively firming scheduled energy, and optimizing energy arbitrage opportunities. Additionally, the test rigorously assesses the software platform's reliability, security, and scalability, ensuring its aptitude for meeting diverse operational demands.

The GEMS IntelliBidder is a key component of Wärtsilä's GEMS digital energy platform, a comprehensive solution designed for the monitoring, control, and optimization of energy assets in hybrid projects at both individual site and broader portfolio levels (Wärtsilä 2022a). Utilizing advanced machine learning algorithms, GEMS IntelliBidder is equipped with capabilities for forecasting market prices and renewable power generation. It manages schedule commitments and optimizes bids across various electricity markets, such as ERCOT and CAISO (Wärtsilä 2022b). One of its significant features is the integration of APIs from ISOs, enabling automated bidding processes that reduce the risk of human error. Additionally, GEMS IntelliBidder supports different trading environments, including day‐ahead markets, real‐time markets, and power purchase agreements.

## 7 Discussion

The literature review underscores the extensive research conducted on the EMS of HPPs. However, numerous challenges persist. Based on the overview detailed in Section 4, much attention has been devoted to the optimal operation of HPPs in energy markets, while ancillary services provided by HPPs remain relatively neglected. It can be foreseen that HPPs participating in ancillary service markets bring challenges and opportunities to the research areas. Ancillary services are essential for balancing supply and demand, managing grid frequency, and providing reserve power, which is critical for the steady operation of energy systems, particularly as energy systems transition towards 100% RES. Yet, gaps persist in this area. For instance, the capability of HPPs to offer balancing services comparable to conventional power plants remains unclear due to uncertainties of renewable generation. Moreover, research focusing on the optimal offering and operational strategies of HPPs in European balancing markets is notably scarce, highlighting a substantial gap that warrants further exploration and investigation. To provide ancillary services, HPPs must reserve power capacity before they know the real renewable generation and ensure the committed power capacity is available in real time. Otherwise, if HPPs cannot deliver the activated power, energy systems might suffer instability, and HPPs might be banned from providing ancillary services and, therefore, lose opportunities to capture the revenue streams. This brings new challenges for the EMS modeling because, apart from the uncertainty of renewable generation, the EMS must also incorporate the uncertainty of ancillary service requirements to make reliable power reserves.

However, as discussed in Section 5, significant efforts have been put into handling the uncertainty of renewable power and market prices when developing HPP EMS, while the uncertainty of ancillary service requirements is lacking attention. Generally, there are two approaches to dealing with uncertainties in EMS: (i) Enhance the forecasting accuracy and (ii) Apply advanced uncertainty handling methodologies. Therefore, developing reliable and robust forecasting methods for ancillary service requirements is an important and emerging research area. However, the ancillary service requirements are highly dependent on the power system operation status, such as generation deficit or surplus, and can vary significantly over time. This information is not readily available for HPP operators, which poses a significant challenge and interest for this topic.

Incorporating additional markets and uncertainties increases the complexity of modeling EMS. For instance, Lawati et al. (2021) reflect that new groups of variables and constraints must be integrated into EMS to accommodate multiple markets. Furthermore, the interaction between newly introduced and existing markets must also be effectively modeled. It has been found that machine learning is becoming a promising technique for addressing this challenge of HPP EMS. Machine learning helps EMS learn from historical data and improve its performance without explicit models. As reviewed in Section 3, DRL has been applied in modeling HPP EMS. For example, the case study by Ochoa et al. (2022) indicates that DRL‐based EMS performs better in helping HPPs in day‐ahead and real‐time markets than traditional model‐based EMS. This superior performance is attributed to DRL's ability to continuously learn and adapt to changing market conditions, optimizing energy distribution more efficiently. Despite these promising developments, there are concerns about the practical applicability of DRL‐based EMS. One significant issue is DRL's dependency on the quality and quantity of available data. The performance of a DRL‐based EMS is directly linked to the data it has been trained on. The DRL may not perform as expected when data is scarce, outdated, or not representative of actual market conditions. This dependency raises questions about the reliability and consistency of DRL‐based EMS in varying operational contexts. Moreover, the “black‐box” nature of DRL models poses another challenge. Unlike traditional models with explicit and transparent decision‐making logic, DRL models often lack interpretability. This lack of transparency can be a barrier in industries where understanding and explaining decision‐making processes is crucial for regulatory compliance and stakeholder trust.

## 8 Conclusion

This paper provides a comprehensive overview of energy management systems (EMS) for grid‐connected, utility‐scale hybrid power plants (HPPs). It offers a detailed look at different HPP configurations, EMS strategies, market and uncertainty considerations, as well as industrial EMS solutions. This paper critically highlights existing challenges and emerging opportunities. While there has been a substantial volume of research, the field remains ripe for further exploration and development. As a key contribution, the paper provides a comprehensive understanding of the development of HPP EMS. The main findings of the review are:

- The integration of wind, photovoltaic, and battery storage to form HPPs has garnered substantial interest in recent research, followed by the combination of wind, photovoltaic, hydropower plants, and concentrated solar plants.
- Mathematical optimization has become the principal approach in modeling EMS for HPPs. This prominence is attributed to its proven effectiveness and the availability of a wide range of optimization models tailored to meet the varied demands of EMS.
- While considerable research has been devoted to the energy markets of HPPs, the role and potential of HPPs in the ancillary service market remains areas ripe for further investigation.
- Studies have extensively covered the uncertainties associated with renewable power generation and market prices. However, the uncertainty in ancillary services also merits focused attention.
- On the industrial front, effective EMS tools are available for HPPs, indicating a harmonious bridge between theoretical advancements and practical implementation.

The future direction of energy management EMS for hybrid power plants is likely to concentrate on integrating advanced forecasting technologies and sophisticated modeling strategies to effectively manage the growing complexity and uncertainty associated with participation in multiple energy markets. One significant area of development could involve enhancing the handling of ancillary service requirements, which are becoming increasingly unpredictable. This could be achieved through innovative forecasting and modeling techniques such as learning‐to‐optimize approaches, which use the performance of EMS as the metric to evaluate the forecast.

## Author Contributions

**Rujie Zhu:** conceptualization (equal), data curation (lead), formal analysis (equal), investigation (equal), methodology (equal), visualization (lead), writing – original draft (lead), writing – review and editing (equal). **Kaushik Das:** supervision (equal), validation (equal), writing – review and editing (equal). **Poul Ejnar Sørensen:** funding acquisition (equal), methodology (equal), supervision (equal). **Anca Daniela Hansen:** funding acquisition (equal), project administration (equal), supervision (equal), validation (equal), writing – review and editing (equal).

## Conflicts of Interest

The authors declare no conflicts of interest.

## Related WIREs Articles

Distributed energy resource management systems‐DERMS: State of the art and how to move forward

## Data Availability

Data sharing is not applicable to this article as no new data were created or analyzed in this study.
